# Biologically active fibroblast growth factor 1 tagged with various epitopes

**DOI:** 10.1186/1756-0500-1-42

**Published:** 2008-07-11

**Authors:** Masahiro Asada, Emi Honda, Toru Imamura

**Affiliations:** 1 Signaling Molecules Research Laboratory, National Institute of Advanced Industrial Science and Technology (AIST), Tsukuba Central #6, 1-1-1 Higashi, Tsukuba, Ibaraki 305-8566, Japan

## Abstract

**Background:**

Fibroblast growth factor (FGF) family members are involved in the regulation of a variety of biological phenomena. Because most of their activity is exerted via a signaling complex composed of FGF, heparin/heparan sulfate and FGF receptor tyrosine kinase, it is important to study the dynamic behavior of all the molecules in the complex without disturbing their interaction or activity.

**Findings:**

We used *E. coli *to express biologically active human FGF1 tagged at its C-terminus with myc-(His)_6_, V5-(His)_6 _or 3xFLAG-(His)_6_. We found that the tagged FGF1s had affinities for heparin that were similar to that of the native form. The tagged FGF1s also exhibited mitogenic activity similar to that of the native form. Apparently, the tags do not interfere with the formation of the three-member complex involving FGF1, FGF receptor and heparan sulfate/heparin.

**Conclusion:**

Tagged FGF1s should be useful for investigating the dynamic behavior of FGF1 in the context of its three-member signaling complex and other molecular complexes.

## Findings

### Background

Epitope tags are frequently introduced when expressing recombinant proteins, as they make it easy to purify and detect proteins of interest. However, addition of exogenous amino acids can alter the properties of proteins, including their affinity for other biomolecules and their biological activities [1]. Nevertheless, the effect of introducing an epitope tag on the activity of a protein of interest has rarely been addressed.

Fibroblast growth factors (FGFs) are multifunctional proteins that play important roles in many biological phenomena. Their effects are elicited mainly through simultaneously binding to various cell surface FGF receptors (FGFRs) and heparan sulfate sugar chains. In order to analyze the structural features of the resultant three-member complexes, we considered it useful to label each member with a different epitope tag. Because such analyses would require biologically active FGF1 to be tagged in various formats, we developed those proteins and report some of their characteristics here.

## Methods

### Construction of cDNAs

**Figure 1:**
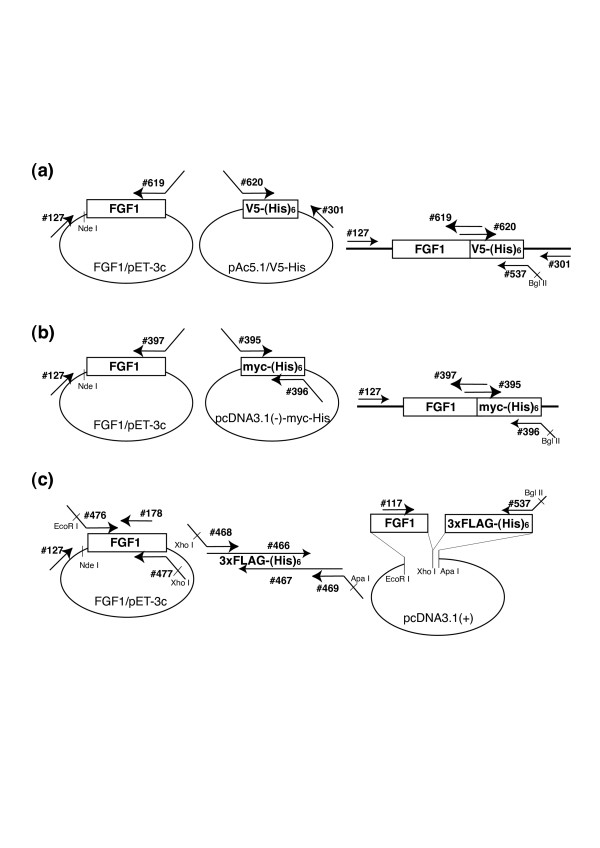
**Schematic diagram depicting the strategy for plasmid construction**. Complementary DNAs encoding FGF1 and V5-(His)_6 _tag (a), myc-(His)_6 _tag (b) or 3xFLAG-(His)_6 _tag (c) were separately amplified, and the products were mixed and used as templates for overlap extension PCR. The sequences of the primers are summarized in Table 1.

The strategy for constructing cDNAs encoding chimeric FGF1s is depicted schematically in Fig. 1, and the primers used are listed in Table 1. Human FGF1 cDNA was cloned into pET-3c vector as described previously [2]. To generate cDNA encoding the chimeric proteins, PCR was carried out using the plasmid as a template with #127 serving as the forward primer and #619 (for V5-(His)_6 _tag) or #397 (for myc-(His)_6 _tag) serving as the reverse primer. To generate the cDNA encoding V5-(His)_6 _tag, pAc5.1/V5-His vector (Invitrogen) served as the template with primers #620 and #301. For cDNA encoding myc-(His)_6 _tag, pcDNA3.1(-)-myc-His vector (Invitrogen) served as the template with primers #395 and #396.

**Table 1:** Primers used in this study

No.	for./rev.	sequence	site
#127	forward	5'-gta ata cga ctc act ata ggg-3'	vector primer
#619	reverse	5'-acc ttc atc aga aga gac tgg cag-3'	FGF1/3' + V5-(His)_6_/5'
#397	reverse	5'-cga gct cgg atc aga aga gac tgg cag-3'	FGF1/3' + myc-(His)_6_/5'
#620	forward	5'-tct gat gaa ggt aag cct atc cct-3'	FGF1/3' + V5-(His)_6_/5'
#301	reverse	5'-tag aag gca cag tcg agg-3'	vector primer
#395	forward	5'-cca gtc tct tct gat ccg agc tcg gta cca-3'	FGF1/3' + myc-(His)_6_/5'
#396	reverse	5'-gct ctA GAT CTt caa tga tga tga tga tga tgg tc-3'	myc-(His)_6_/3' + Bgl II
#468	forward	5'-cag ccg CTC GAG a-3'	Xho I + 3xFLAG-(His)_6_/5'
#469	reverse	5'-tgc GGG CCC tca a-3'	3xFLAG-(His)_6_/5' + Apa I
#466	forward	5'-ccg CTC GAG act aca aag acc atg acg gtg att ata aag atc atg aca tcg act aca ag-3'	megaprimer/5'
#467	reverse	5'-tgc GGG CCC tca atg gtg atg gtg atg atg acc ctt gtc atc gtc atc ctt gta gtc ga-3'	megaprimer/3'
#476	forward	5'-cgG AAT TCc cac cat gtc ccg ggg agc-3'	EcoR I + FGF1/5'
#477	reverse	5'-ccg CTC GAG cat cag aag aga ctg gca g-3'	FGF1/3' + Xho I
#117	forward	5'-tct tcc gat aga ctg cgt cg-3'	FGF1
#537	reverse	5'-gaA GAT CTc ttc aat ggt gat ggt gat gat gac c-3'	(His)_6_/3' + Bgl II
#178	reverse	5'-agc ccg tcg gtg tcc atg gc-3'	FGF1

Note that the capital letters indicate the recognition sequence of restriction enzymes.

To create a cDNA encoding 3xFLAG-(His)_6_, PCR was carried out with primers #468 and #469 in the presence of two megaprimers, #466 and #467, which served as the template. The product was digested with Xho I and Apa I and cloned into predigested pcDNA3.1(+), yielding plasmid 3xFLAG-(His)_6_/pcDNA3.1(+). The open reading frame for FGF1 was then amplified using #476 and #477, digested with EcoR I and Xho I, and inserted into predigested 3xFLAG-(His)_6_/pcDNA3.1(+). Using this plasmid as a template, PCR was then carried out with primers #117 and #537 to generate cDNA encoding the 3'-part of FGF1-3xFLAG-(His)_6_. For the 5'-part, PCR was carried out using FGF1/pET-3c as template with primers #127 and #178.

Once the first round PCR products were purified, overlap extension PCR was carried out to generate the intended cDNAs encoding the chimeric FGF1s. For FGF1-V5-(His)_6_, the PCR products of #127/#619 and #620/#301 were mixed as templates, and second round PCR was carried out using primers #127 and #537. In the same way, for FGF1-myc-(His)_6_, the products of #127/#397 and #395/#396 were mixed as the template, and second round PCR was carried out using primers #127 and #396. For FGF-1-3xFLAG-(His)_6_, the PCR products of #127/#178 and #117/#537 were mixed as the template, and second round PCR was carried out using primers #127 and #537.

Once the full-length cDNAs encoding the chimeric proteins were generated, the DNA for the open reading frame was excised using Nde I/Bgl II and ligated into pET-3c vector (Novagen) predigested with Nde I/BamH I. The cDNA sequences were verified to be the intended sequences using an ABI310 genetic analyzer. The expected numbers of amino acid residues for native FGF1, FGF1-myc-(His)_6_, FGF1-3xFLAG-(His)_6 _and FGF1-V5-(His)_6 _were 136, 165, 167 and 160, respectively.

### Expression and semipurification of the recombinant FGF1

Expression vectors generated as described above were used to transform *Escherichia coli *strain BL21(DE3)/pLysS cells (Novagen), after which positive clones were selected. After preincubating the cells for 3 h at 37°C, protein expression was induced by addition of IPTG (final concentration: 1 mM), and the incubation was continued for an additional 4 h at 37°C. The cells were then collected by centrifugation and suspended in GET buffer (10 mM glucose, 10 mM EDTA, 50 mM Tris-HCl, pH 7.4). The cell walls of the suspended cells were disrupted by freeze/thaw and sonication, after which the resultant lysate was filtered (0.45 μm), and the salt concentration was adjusted to 0.5 M. Heparin-Sepharose (Amersham Bioscience) beads were then added, and the mixture was incubated for 18 h at 4°C. The resin was then packed into a small column and washed extensively with wash buffer (10 mM Tris-HCl, pH 7.4, 500 mM NaCl), after which the bound protein was eluted with elution buffer (10 mM Tris-HCl, pH 7.4, 2.0 M NaCl). The peak fractions (judged from the absorbance at 280 nm) were combined and subjected for further analysis.

### Gel electrophoresis and immunoblot analysis

Semipurified recombinant FGF1s were resolved by SDS-PAGE (12.5%) and stained with Coomasie brilliant blue (CBB) or transferred to nitrocellulose membranes (Schleicher & Schuell BioScience Inc.) for immunostaining. The membranes were probed with monoclonal anti-FGF1 (mAb1; 1 μg/ml) [3], anti-myc (1/5,000 dilution; Invitrogen), anti-FLAG (M2; 10 μg/ml; Sigma) or anti-V5 (1/2,000 dilution; Invitrogen) antibodies in Tris-buffered saline (TBS: 10 mM Tris-HCl, 0.15 M NaCl, pH 7.4) containing 5% skim milk (SM), or monoclonal anti-His antibody (anti-penta-His; 0.2 μg/ml; QIAGEN) in TBS containing 3% bovine serum albumin (BSA). HRP-conjugated goat anti mouse IgG (1/10,000 dilution; Zymed) in TBS with 5% SM served as the secondary antibody for all primary antibodies except anti-His. For anti-His, the same secondary antibody (1/10,000 dilution) was used in TBS with 5% BSA. The blots were visualized using an ECL western blotting detection system (Amersham Bioscience) according to the manufacturer's instructions.

### Heparin affinity analyzed with HPLC

The affinities of the semipurified proteins for heparin were analyzed using a Toso-HPLC system equipped with a TSKguardgel Heparin-5PW column (6 mm ID × 1 cm). Tris-HCl buffer (pH 7.4) containing 0.15 M NaCl (buffer A) or 2.0 M NaCl (buffer B) were used as the effluents, the flow rate was 1.0 ml/min, and the eluate was fractionated into 0.5-ml fractions. The elution profile was monitored based on the absorbance at 280 nm. The salt concentration of each fraction was calibrated based on its conductivity.

Aliquots of the HPLC fractions were subjected to dot-blot analysis. Samples were loaded onto a nitrocellulose filter in a BioRad dot blotting system and then blocked with 5% SM. As the antibody mAb1 preferentially binds to denatured FGF1, the filter was first incubated in 2% SDS and 100 mM 2-mercaptoethanol for 30 min at 70°C, after which is was blocked in 5% SM again and immunostained using mAb1 as described above.

### Assaying the mitogenic activity of FGF1

Ba/F3 cells were purchased from RIKEN. An expression vector encoding the FGFR1-IIIc was a generous gift from Dr. D. M. Ornitz (Washington Univ., St. Louis, MO). The vector was transfected into Ba/F3 cells by electroporation, after which neo-resistant clones were selected. Ba/F3 cells expressing FGFR1-IIIc (designated FGFR1c-Ba/F3 cells) were cultivated in RPMI1640 supplemented with 10% fetal bovine serum, 50 ng/ml recombinant FGF1, 5 μg/ml heparin and 0.5 mg/ml geneticin. To assay mitogenic activity, FGFR1c-Ba/F3 cells were seeded into a microplate to a density of 6.6 × 10^3 ^cells/well and incubated with the indicated amount of each tagged FGF1 and 10% FCS in the presence or absence of heparin (10 μg/ml). After 48 h, TetraColor ONE (Seikagaku Kogyo, Tokyo) was added, and the cells were incubated for an additional 4 h, after which the absorbance at 450 nm was measured.

## Results and discussion

### Construction and expression of tagged FGF1

**Figure 2:**
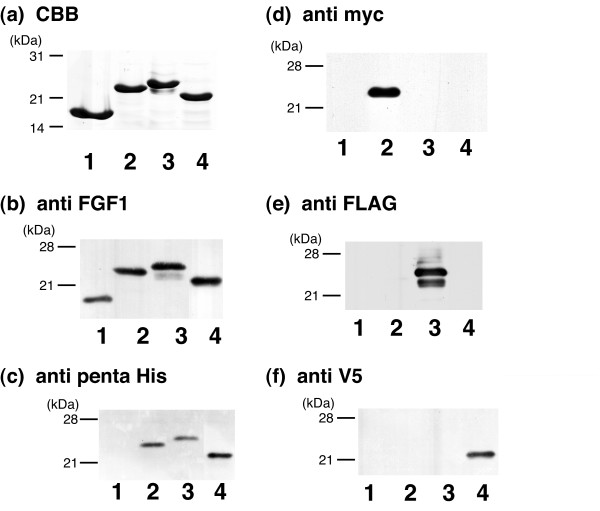
**Electrophoresis and immunoblot analysis of the expressed proteins**. FGF1 (lane 1), FGF1-myc-(His)_6 _(lane 2), FGF1-3xFLAG-(His)_6 _(lane 3) and FGF1-V5-(His)_6 _(lane 4) were expressed by *E. coli *and semipurified by heparin-Sepharose affinity chromatography. The eluate was subjected to SDS-PAGE and stained with CBB (a). After electrophoresis, the separated proteins were transferred to nitrocellulose membranes and immunoblotted using monoclonal anti-FGF1 (b), anti-His (c), anti-myc (d), anti-FLAG (e) or anti-V5 (f) antibodies. Positions of the molecular weight markers are indicated at the left of each panel in kDa.

Using overlap extension PCR, we constructed a set of cDNAs encoding FGF1 with various tags. The proteins expressed in *E. coli *had the expected molecular weights and were semipurified by heparin affinity column chromatography. Subsequent SDS-PAGE revealed that each construct was purified to homogeneity, as indicated by the single band after CBB staining (Fig. 2a). A monoclonal anti-FGF1 antibody (mAb1) recognized all of the proteins expressed (Fig. 2b). By contrast, monoclonal antibodies against (His)_6_-tag, myc-tag, FLAG-tag and V5-tag specifically recognized the correspondingly tagged FGF1, but not FGF1s with different tags (Fig. 2c–f). The minor band accompanying FGF1-3xFLAG-(His)_6 _detected with anti-FLAG antibody (Fig. 2e) is likely a partially N-truncated fragment of the intact protein, since the tag is at the C-terminus.

### Heparin-affinity of the tagged FGFs

**Figure 3:**
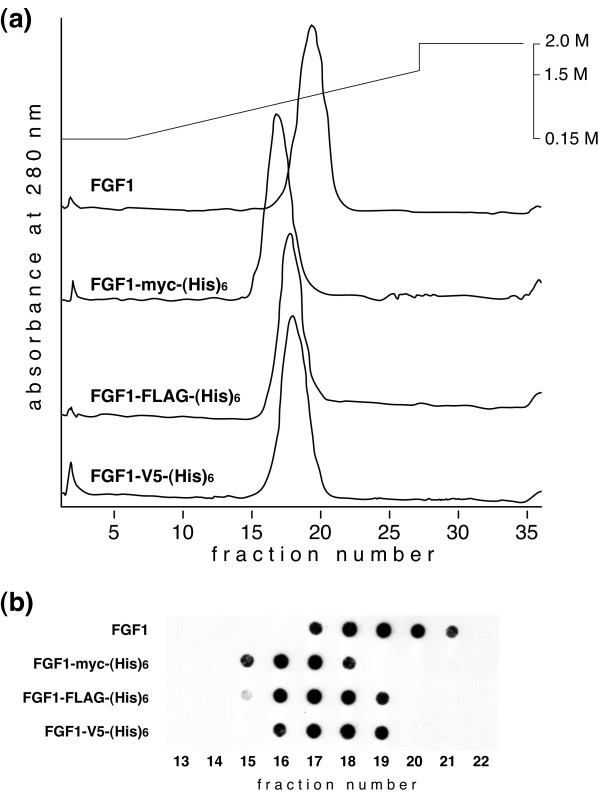
**HPLC analysis of the affinity of semipurified FGF1s for heparin**. The elution profiles of FGF1, FGF1-myc-(His)_6_, FGF1-3xFLAG-(His)_6 _and FGF1-V5-(His)_6 _are illustrated in (a). NaCl concentration was gradually increased as illustrated at the top, and the absorbance at 280 nm was monitored. An aliquot of the peak fraction was also subjected for dot-blot analysis using anti-FGF1 monoclonal antibody (b).

To determine their affinity for heparin, batch-purified FGF1s were subjected to heparin affinity HPLC and eluted with a linear NaCl gradient. A single absorbance peak at 280 nm was detected for each tagged FGF1 (Fig. 3a), and the FGF1 constructs in the peak fractions were confirmed by dot blot analysis using monoclonal anti-FGF1 antibody (Fig. 3b). We found that FGF1, FGF1-myc-(His)_6_, FGF1-3xFLAG-(His)_6 _and FGF1-V5-(His)_6 _were eluted from heparin Sepharose with 1.24, 1.09, 1.17 and 1.17 M NaCl, respectively, which is in good agreement with earlier reports that the NaCl concentration required for FGF1 elution from heparin is 1–1.2 M NaCl [4].

Because Lacy and Sanderson previously reported that (His)_6 _tag enhanced the affinity of Sp17 protein for heparan sulfate [5], we analyzed the effect of various tags on the affinity of FGF1 protein for heparin. FGF1 has a strong affinity for heparin/heparan sulfate, and the interaction may be critically important for its biological activity. We found that the myc-(His)_6_, 3xFLAG-(His)_6 _and V5-(His)_6 _tags used in this study had little effect on the affinity of FGF1 for heparin. Given that the three-dimensional structure of FGF1 indicates that its N- and C-termini are flexible and outside the β-barrel structure important for receptor binding and heparin affinity [6], it is likely that addition of a tag at the C-terminus has no effect on the protein's affinity to heparin/heparan sulfate.

### Mitogenic activity of tagged FGF1

**Figure 4:**
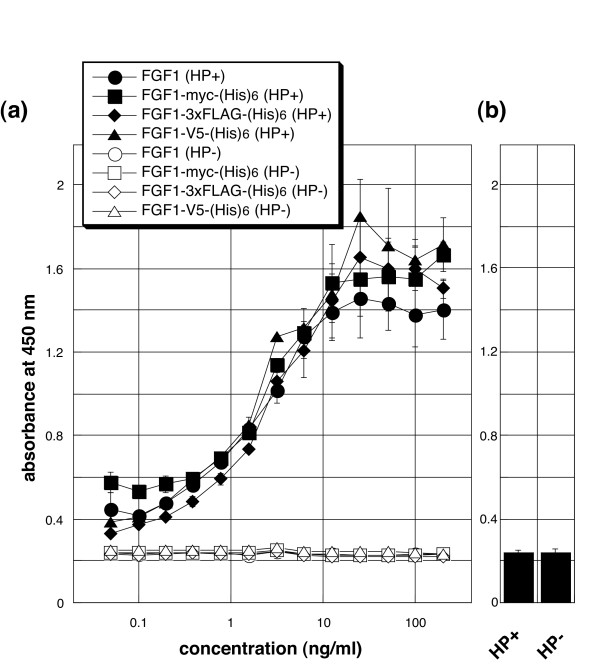
**Mitogenic activity of FGF1s towards FGFR1c-Ba/F3 cells**. (a) FGFR1c-Ba/F3 cells were cultivated for 48 h with the indicated concentrations of FGF1s in the presence of 10 μg/ml heparin (closed symbols). TetraColor ONE was then added, and the cells were incubated for an additional 4 h, after which the absorbance at 450 nm was measured. No mitogenic activity was seen in the absence of heparin (open symbols). (b) In the absence of the FGF1s, no activity was observed, despite the presence of heparin. The data presented are the averages and standard deviations of a triplicate experiment. Independent experiments were performed twice, and essentially the same results were obtained.

We also investigated the ability of tagged FGF1 to stimulate proliferation of FGFR1c-Ba/F3 cells [7]. As shown in Fig. 4a, the mitogenic activities of FGF1s tagged with myc-(His)_6_, 3xFLAG-(His)_6 _or V5-(His)_6 _were indistinguishable from those of native FGF1. Moreover, statistical analysis revealed no significant difference between any of the FGF1s. These results strongly suggest that in the presence of heparin these tagged FGF1s have affinities for cell surface FGFR1-IIIc that are similar to native FGF1. In addition, like native FGF1, they exhibited no activity in the absence of heparin, nor was any mitogenic activity detected in the absence of FGF1, despite the presence of heparin (Fig. 4b).

We have shown here that introduction of a myc-(His)_6_, 3xFLAG-(His)_6 _or V5-(His)_6 _tag at the C-terminus of FGF1 had little or no effect on the growth factor's affinity for heparin and its mitogenic activity toward cells expressing FGFR1-IIIc. Apparently, FGF1, FGFR and heparin are able to form an active signaling complex on the cell surface, despite the presence of a C-terminal tag. These tagged FGF1s should be useful for investigating the dynamic behavior of FGF1 in the context of its three-member signaling complex and other molecular complexes.

## Competing interests

The authors declare that they have no competing interests.

## Authors' contributions

TI conceived the study design, was responsible for the data collection, contributed to the data analysis and the writing of the paper. MA completed analyses, interpreted findings, and was the principal author of the manuscript. EH was involved in the study design, contributed to the interpretation of data, and editing of the paper.
